# Chronic Epinephrine-Induced Endoplasmic Reticulum and Oxidative Stress Impairs Pancreatic β-Cells Function and Fate

**DOI:** 10.3390/ijms25137029

**Published:** 2024-06-27

**Authors:** Ran Zhang, Bingpeng Yao, Rui Li, Sean W. Limesand, Yongju Zhao, Xiaochuan Chen

**Affiliations:** 1 College of Animal Science and Technology, Southwest University, Chongqing 400715, China; 18375628026@163.com (R.Z.); ybp9620@163.com (B.Y.); lirui_214@163.com (R.L.); zyongju@163.com (Y.Z.); 2 School of Animal and Comparative Biomedical Sciences, The University of Arizona, Tucson, AZ 85721, USA; limesand@arizona.edu

**Keywords:** pancreatic β-cells, epinephrine, ER stress, oxidative stress, α2A-adrenergic receptor

## Abstract

Epinephrine influences the function of pancreatic β-cells, primarily through the α2A-adrenergic receptor (α2A-AR) on their plasma membrane. Previous studies indicate that epinephrine transiently suppresses insulin secretion, whereas prolonged exposure induces its compensatory secretion. Nonetheless, the impact of epinephrine-induced α2A-AR signaling on the survival and function of pancreatic β-cells, particularly the impact of reprogramming after their removal from sustained epinephrine stimulation, remains elusive. In the present study, we applied MIN6, a murine insulinoma cell line, with 3 days of high concentration epinephrine incubation and 2 days of standard incubation, explored cell function and activity, and analyzed relevant regulatory pathways. The results showed that chronic epinephrine incubation led to the desensitization of α2A-AR and enhanced insulin secretion. An increased number of docked insulin granules and impaired Syntaxin-2 was found after chronic epinephrine exposure. Growth curve and cell cycle analyses showed the inhibition of cell proliferation. Transcriptome analysis showed the occurrence of endoplasmic reticulum stress (ER stress) and oxidative stress, such as the presence of *BiP*, *CHOP*, *IRE1*, *ATF4*, and *XBP*, affecting cellular endoplasmic reticulum function and survival, along with *UCP2*, *OPA1*, *PINK*, and *PRKN*, associated with mitochondrial dysfunction. Consequently, we conclude that chronic exposure to epinephrine induces α2A-AR desensitization and leads to ER and oxidative stress, impairing protein processing and mitochondrial function, leading to modified pancreatic β-cell secretory function and cell fate.

## 1. Introduction

Type 2 diabetes mellitus (T2DM) is a chronic metabolic disease characterized primarily by elevated blood glucose. Insulin, as the only hormone in the body that lowers blood glucose, is essential for glucose metabolism and overall metabolic balance. T2DM occurs when pancreatic β-cells fail to release enough insulin to meet the demands of insulin-responsive tissues [1]. In addition to insulin resistance in the peripheral tissues, β-cell dysfunction is also a major characteristic in T2DM [2]. These conditions are usually caused by poor lifestyle habits, unbalanced diet, and a lack of exercise [3]. Likewise, a regular physical activity routine, coupled with a well-balanced nutritional intake, positively affects individuals suffering from diabetes [4]. Accumulating evidence suggests that pancreatic β-cells exhibit significant plasticity during the development of T2DM [5], and that under normal physiological conditions, pancreatic β-cells can cope with short-term stress through a range of adaptive responses, but when they exceed a certain threshold, β-cell properties are progressively lost [6,7].

Multiple hormonal factors influence β-cell responsiveness. Chronic stress leads to the disruption of the hypothalamic–pituitary–adrenal (HPA) axis balance, triggering an overabundance of stress hormones such as glucocorticoids and catecholamine [8,9]. This hormonal imbalance has been observed to negatively impact the functionality of β-cells across various experimental settings, including in vitro insulin-secreting cell lines, rodent and human pancreatic islets in culture, and in vivo rodent and ovine models [10,11]. The secretion of catecholamines, while influenced by both the nervous system and the adrenal glands, is predominantly driven by the adrenal glands, as confirmed by adrenalectomy studies [12]. It is significant to note that catecholamines are pivotal in the body’s immediate response to chronic stress, acting as the initial signaling molecules that regulate the stress response.

Adrenergic receptors are activated by catecholamines, such as epinephrine and norepinephrine, which are triggered during exercise or stress. Usually, catecholamines attenuate β-cell activity and inhibit insulin release by activating α2-adrenergic receptors on the plasma membrane of the β-cells, mainly by decreasing the production of cAMP and by having a desensitizing effect on intracellular Ca^2+^ concentration during the later stages of the cytotoxic process [13]. A recent study has shown that elevated fetal catecholamines are associated with neonatal β-cell physiology, suggesting a mechanistic link between prenatal or perinatal stress and subsequent neonatal hyperinsulinemic hypoglycemia [14]. This is consistent with findings that injection of norepinephrine into fetal sheep for 7 consecutive days resulted in a compensatory enhancement of insulin secretion [11]. In addition, MIN6 (mouse insulinoma 6) showed differential expression of metabolic-related proteins after acute adrenergic stimulation [15]. In previous reports, chronic adrenergic stimulation the lower *UCP2* abundance of β-cells and induced ROS accumulation, which contributed to oxidative damage and cytotoxicity [10,15]. But how adrenergic receptors impact β-cell survival is still unknown.

Survival is another important aspect of pancreatic β-cell function, as it determines the β-cell mass and the ability to compensate for insulin resistance. There are multiple transcription factors (PDX1, MafA, and NeuroD1) and growth factors, (IGF-1 and VEGF) that regulate β-cell development, function, and survival [16]. Moreover, cellular senescence is closely related to cellular stresses, including ER stress, which causes β-cell dysfunction [17]. Pancreatic β-cells experience high levels of ER stress due to their role in insulin secretion [18]. In order to relieve ER stress, the cells activate the unfolded protein response (UPR), which enhances ER function. However, prolonged or excessive ER stress can trigger apoptosis through the activation of transcription factors such as CHOP and ATF4, as well as the induction of pro-apoptotic proteins such as BIM and BAX [19]. In conclusion, although mild to moderate ER stress promotes β-cell proliferation and insulin synthesis through UPR activation, persistent insulin demand and insulin resistance may lead to excessive ER stress, triggering β-cell dysfunction and apoptosis [20]. To systematically explore the effects of chronic adrenergic signaling exposure on β-cell survival, in this study, we measured insulin secretion, cell proliferation, and apoptosis in MIN6 cells. We also evaluated cellular ultrastructure and analyzed associated regulatory mechanisms. Understanding the impact of adrenergic signaling on β-cell survival will provide important knowledge to find specific targets to resolve the issue.

## 2. Results

### 2.1. Glucose Stimulated Insulin Responsiveness

**Figure 1:**
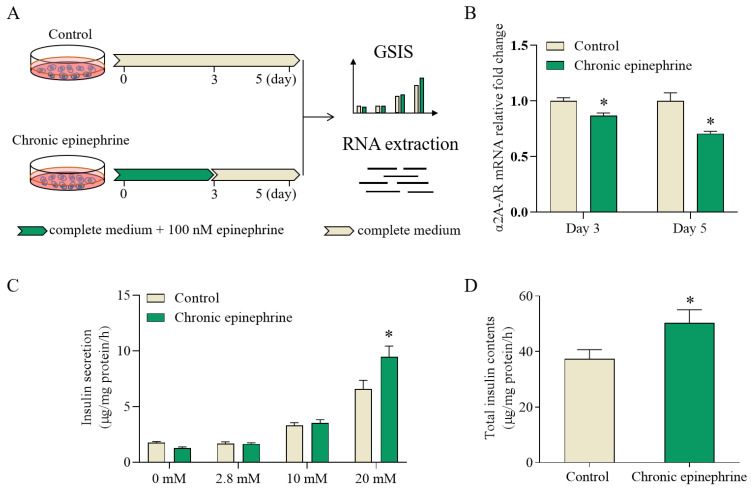
Chronic epinephrine exposure increases insulin secretion responsiveness in MIN6 cells. (**A**) Schematic illustration of the experimental design (n = 4). (**B**) Detection of *α2A-AR* in MIN6 cells on days 3 and 5 by RT-qPCR (n = 4). (**C**) Glucose-induced insulin secretion from MIN6 cells incubated at 0, 2.8, 10, and 20 mM of glucose for 1 h (n = 4). (**D**) Total insulin content of MIN6 secreted after chronic epinephrine incubation (n = 4). * *p* < 0.05.

MIN6 cells were cultured in stimulatory concententrations of epinephrine for 3 days followed by 2 days of epinephrine withdrawal (Figure 1A). The expression levels of *α2A-AR* were measured on the third and fifth day of the experiment, and glucose-stimulated insulin secretion (GSIS) was assessed on the fifth day. Despite removal of epinephrine, *α2A-AR* RNA expression did not show reversible features (Figure 1B). In response to 20 mM glucose, the absolute insulin released into the media increased by 33% (Figure 1C) and insulin content increased by 43% (Figure 1D). These results indicate that MIN6 cells exhibit a persistent state of insulin hypersecretion, even when epinephrine is withdrawn.

### 2.2. Cell Proliferation and Viability

**Figure 2:**
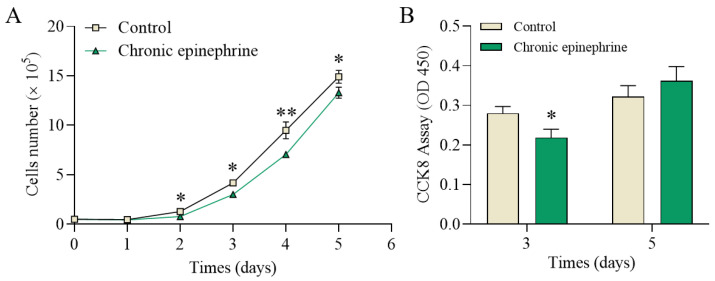
Chronic epinephrine stimulation slowed MIN6 cell proliferation. (**A**) Cell counts for days 1–5 (n > 4). (**B**) Cell Counting Kit 8 (CCK8) assay showing cell viability (n = 4). * *p* < 0.05; ** *p* < 0.01.

Cell numbers were measured each day, and were significantly reduced in the epinephrine-exposed group compared to the control group (Figure 2A). The cell viability assay also showed that the cell proliferation rate was low after epinephrine exposure, although there was no difference on day 5 (Figure 2B).

### 2.3. Differential Gene Expression of RNA-Seq

**Figure 3:**
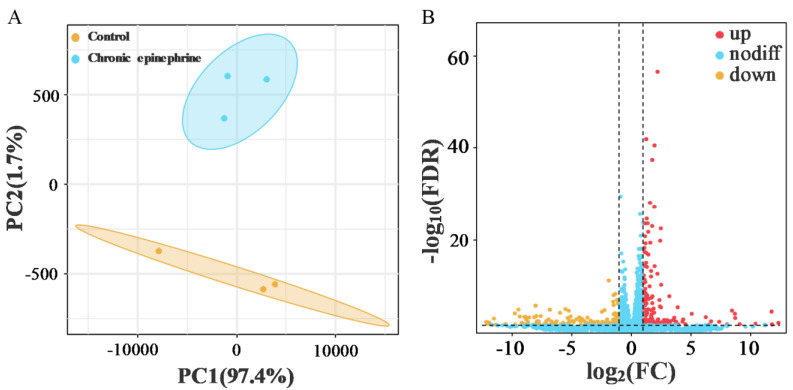
(**A**) PCA score plot representing the difference between the control and chronic epinephrine exposure. (**B**) Volcano plot of global gene expression. The statistically significant genes with a ≥2.0-fold change and a false discovery rate of less than 0.05 are plotted in red (upregulated genes) and yellow (downregulated genes). FDR, false discovery rate; FC, fold change.

**Figure 4:**
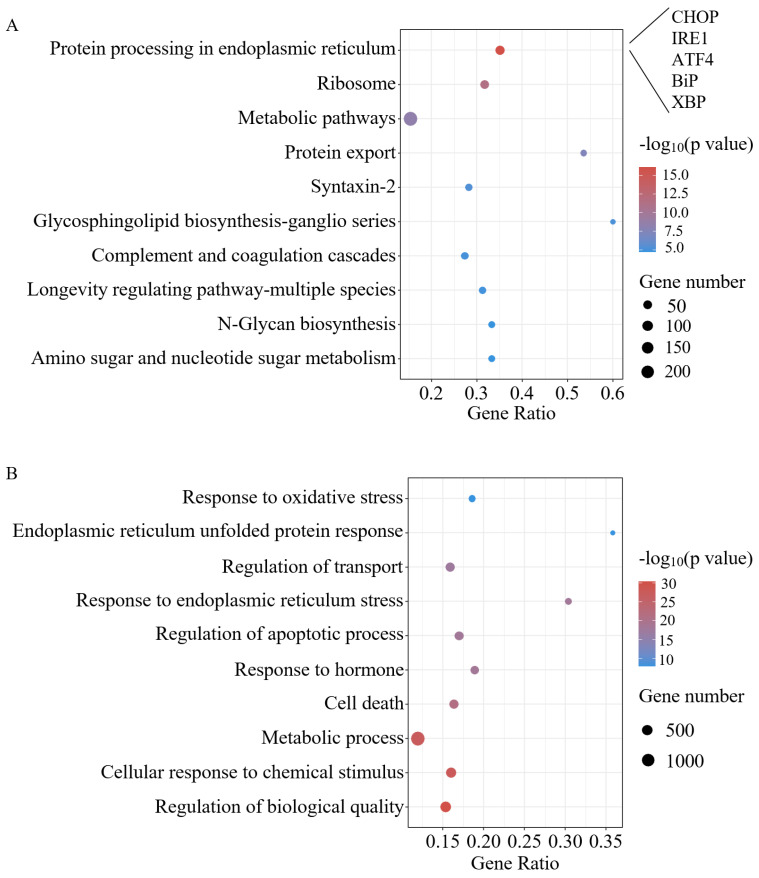
KEGG and GO enrichment analysis. (**A**) Top 10 KEGG enrichment pathway annotation classification results (*p* value < 0.05). (**B**) GO enrichment analysis of biological processes. KEGG, Kyoto Encyclopedia of Genes and Genomes; GO, Gene Ontology.

PCA plots showed good intra-group reproducibility and clear inter-group differences, and volcano plots illustrated that a total of 125 differentially expressed genes (DEGs) were identified, of which 86 genes were upregulated and 39 genes were downregulated, compared with the control group (Figure 3B). These changes in gene expression may involve multiple biological processes in the response of the MIN6 cells to epinephrine. Further differential gene pathway enrichment analysis showed (Figure 4A,B) that enriched KEGG pathways were related to the insulin secretion process, such as endoplasmic reticulum protein processing (e.g., *CHOP*, *IRE1*, *ATF4*, *BiP*, and *XBP*), ribosome production, protein export, Syntaxin-2 health, etc., and GO was mainly enriched in regards to cell fate and oxidative stress-related pathways, such as the response to ER stress, the response to oxidative stress, and the regulation of the apoptotic process.

### 2.4. Impaired Mitochondria Signaling

**Figure 5:**
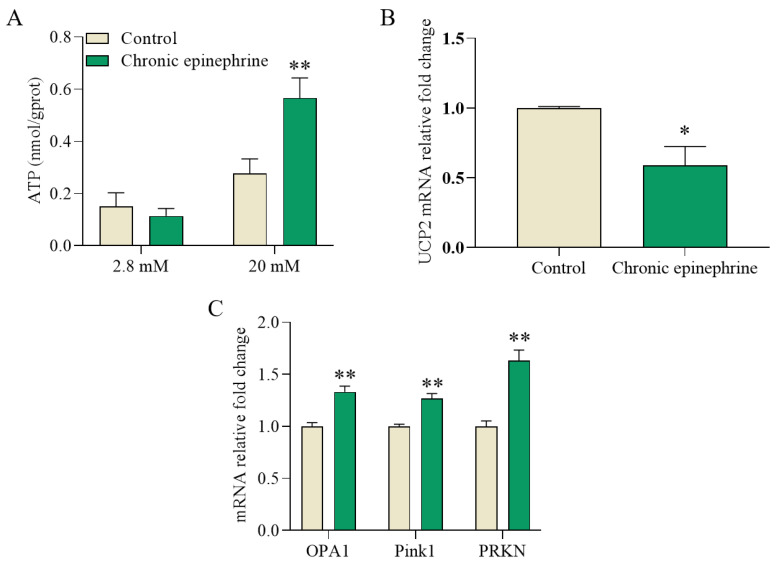
Glucose stimulated increased ATP synthesis and the expression of mitochondria-related genes after chronic epinephrine exposure. (**A**) Cellular ATP concentrations in response to 20 mM glucose (n = 9). (**B**) The gene expression level of *UCP2* in MIN6 cells (n = 4). (**C**) Expression of genes related to mitochondrial dynamics (n = 4). * *p* < 0.05; ** *p* < 0.01.

The GO enrichment analysis identified metabolic processes, which subsequently showed that the levels of ETC and ATP synthase subunits were upregulated, indicating a disruption in mitochondria ATP synthesis (Figure 4B). ATP concentrations and mitochondria-related gene expression were measured in the MIN6 cells on the fifth day. Under high-glucose stimulation, ATP concentrations doubled (Figure 5A). In addition, *OPA1*, *Pink1*, and *PRKN* gene expression levels were upregulated by 32%, 26%, and 62%, respectively, and *UCP2* expression was reduced by 42% (Figure 5B,C).

### 2.5. Ultrastructural Observation of MIN6

**Figure 6:**
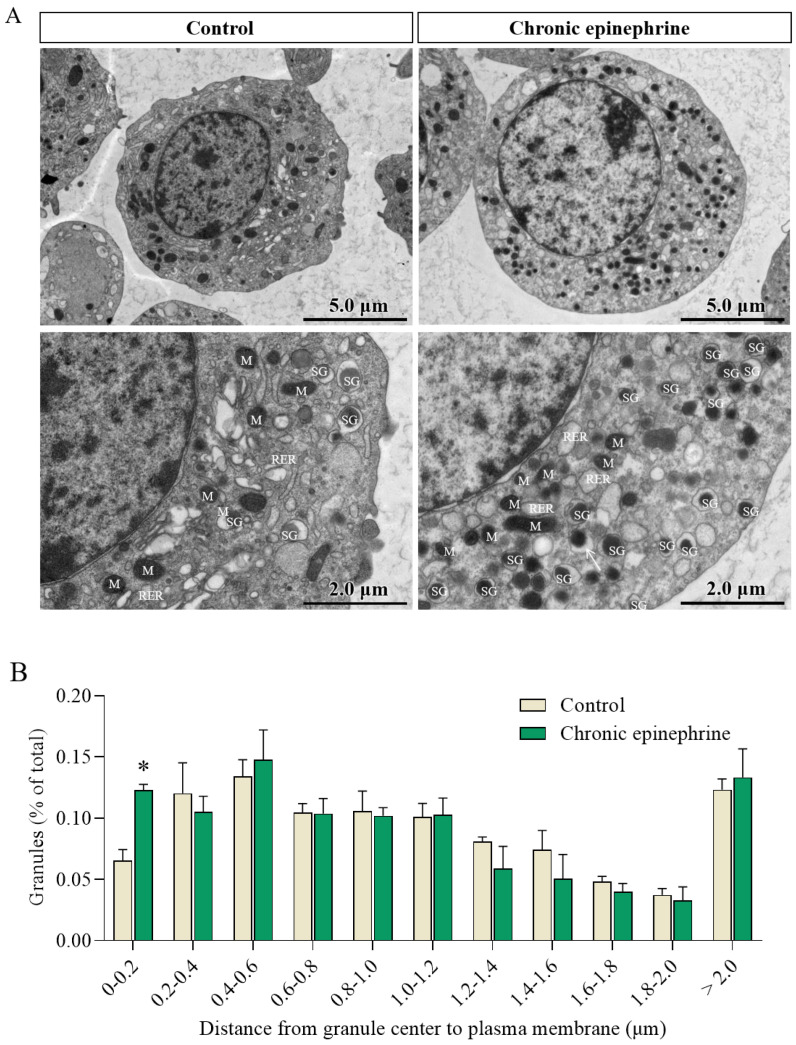
Transmission electron microscopy (TEM) images of MIN6 cells. (**A**) Ultrastructure of insulin granules (SG), mitochondria (M), and rough endoplasmic reticulum (RER). Scale bar = 2 μm. (**B**) The histograms delineate the distribution of insulin granules, derived from an analysis of their relative positioning in the vicinity of the cell membrane using TEM images (n = 3). * *p* < 0.05.

Transmission electron microscopy ultrastructural analysis showed that MIN6 cells exhibited hyperfunction after exposure to epinephrine, with marked dilation of the rough endoplasmic reticulum (RER), many flocculent aggregates visible in the endoplasmic reticulum (ER) pools, and insulin granules of variable size (Figure 6A), with a dense core spilling out (Figure 6A, white arrow). In contrast, the control cells were in an active state, with more insulin granules of uniform size. The counting of insulin granules revealed a significant increase in the number of docked insulin granules after exposure to epinephrine (Figure 6B).

### 2.6. Cell Fate Signaling Pathway

**Figure 7:**
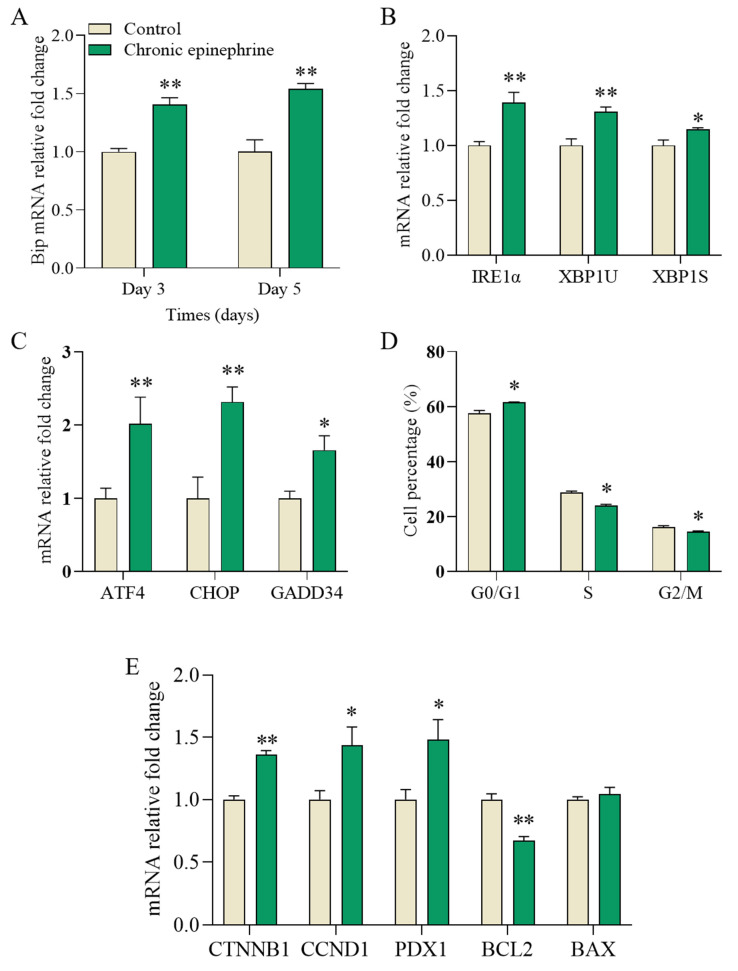
mRNA expression levels and the cell cycle in MIN6 cells. (**A**) Heavy-chain binding protein (*BiP*). (**B**) Inositol-requiring enzyme 1α (*IRE1α*) and X-box binding protein 1 (*XBP1u*, *XBP1s*). (**C**) Activating transcription factor 4 (*ATF4*), C/EBP homologous protein (*CHOP*), and growth arrest and DNA damage-inducible protein (*GADD34*). (**D**) Cell cycle detection by flow cytometry. (**E**) Expression levels of genes associated with proliferation in MIN6 cells (*CTNNB1*, *CCND1*, and *PDX1*); apoptosis in MIN6 cells (*BCL2*, *BAX*). These data were obtained from four biological replicates (n = 4). * *p* < 0.05; ** *p* < 0.01.

The expression of *BiP* was upregulated by 40% on day 3 and by 55% on day 5 compared to that of the control group (Figure 7A). Additionally, the expression of *IRE1α* and its downstream signal transducers *XBP1u* and *XBP1s* were upregulated by 39%, 31%, and 15%, respectively (Figure 7B), while the downstream signals of *PERK*, *ATF4*, *CHOP*, and *GADD34* were upregulated by 101%, 131%, and 65%, respectively (Figure 7C). Cell cycle analysis showed a 4% increase in the percentage of cells in the G0/G1 phase and a decrease in the percentage of cells in the S and G2/M phases (Figure 7D). *CTNNB1*, *CCND1*, and *PDX1* were associated with cell proliferation, which was upregulated by 36% and 44%, respectively; *BCL2* and *BAX* were associated with apoptosis, with *BCL2* downregulated by 33% and *BAX* showing no significant change.

### 2.7. Impaired ROS Regulation

**Figure 8:**
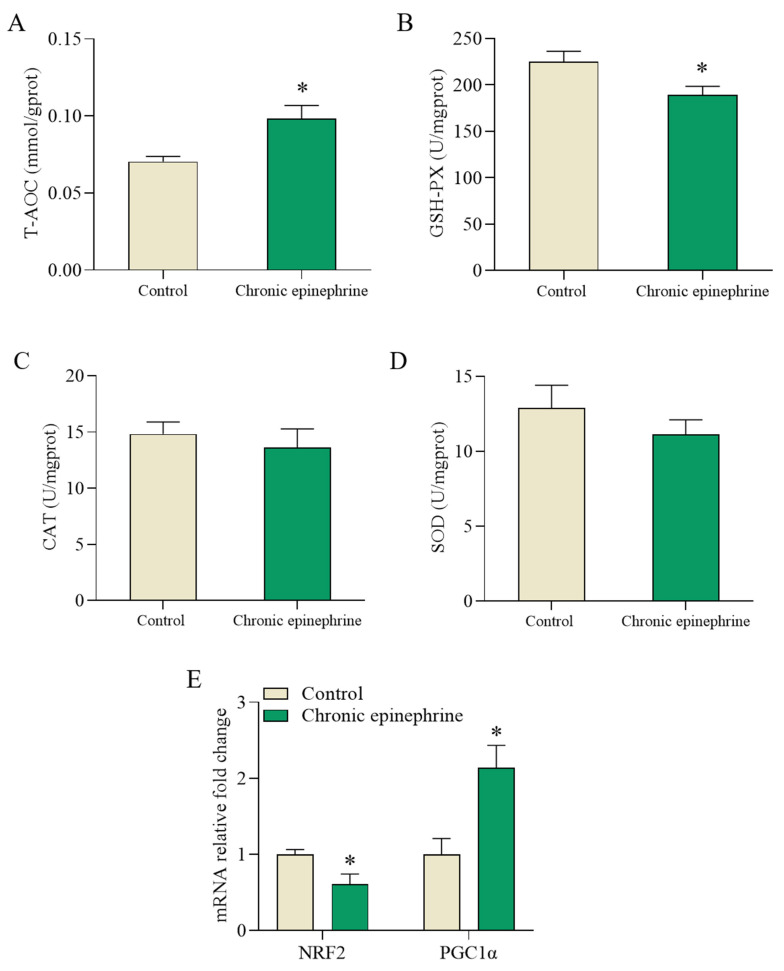
Adaptive response of MIN6 cells to oxidative stress. (**A**) Superoxide dismutase (SOD) activity. (**B**) Glutathione peroxidase (GSH-Px) activity. (**C**) Catalase (CAT) activity. (**D**) Total antioxidant capacity (T-AOC). (**E**) Changes in *NRF2* and *PGC1α* gene expression after chronic epinephrine exposure in MIN6 cells. These data were obtained from at least four biological replicates. * *p* < 0.05.

Compared to that of the control cells, the total antioxidant capacity (T-AOC) was increased by about 66% (Figure 8A), glutathione peroxidase (GSH-Px) activity was decreased by about 21% (Figure 8B), and superoxide dismutase (SOD) and catalase (CAT) activities were unchanged (Figure 8C,D). The expression level of *NRF2*, a gene associated with regulating the expression of antioxidant enzymes, decreased by 38%, whereas the expression level of *PGC1α* doubled (Figure 8E).

## 3. Discussion

Previous studies have demonstrated that chronic adrenergic receptor stimulation has a significant effect on pancreatic β-cell function. In contrast to the acute epinephrine response mechanism, insulin secretion decreased rather than increased when MIN6 cells were exposed to epinephrine for 72 h [21,22], This indicates that prolonged exposure to stimulatory concentrations of epinephrine increases the total insulin content, but the desensitization of adrenergic receptors has not previously been observed [10]. This study further illustrates that prolonged exposure to epinephrine enhances the GSIS capacity of MIN6 cells. This enhancement was sufficient to counteract the inhibitory effect of adrenaline on insulin secretion. Remarkably, even after removal of epinephrine and continued incubation for two days, the MIN6 cells still showed compensatory enhancement (Figure 1C,D). This finding indicates that the effects of this compensatory mechanism may be beyond the range of conventional responses and cannot be reversed by a simple recovery process.

Adrenergic signaling is also an important factor contributing to β-cell dysfunction [23], consistent with previous findings that desensitization of its receptor, α2A-AR, occurs in the presence of prolonged stimulation [22]. At the same time, we still observed a lower *α2A-AR* expression after the withdraw of epinephrine (Figure 1B). α2A-AR, as a G-protein-coupled receptor, normally inhibits insulin release by inhibiting cAMP production [24], but desensitization of α2A-AR in response to prolonged adrenergic action may lead to a diminution of this inhibitory effect, possibly through *UCP2* altering the ATP/ADP ratio (Figure 5B), which in turn increases insulin secretion in a high-glucose environment.

Insulin hyper-secretion responsiveness as an adaptive compensatory mechanism in pancreatic β-cells leads to an increased burden of protein synthesis at the cellular interior, which causes ER stress [25]. As expected, we observed that the rough endoplasmic reticulum of the cells underwent expansion and spillover of the dense core (Figure 6A), and the occurrence of ER stress was confirmed by detecting the level of the ER stress marker *BiP* (Figure 7A) [26]. When ER stress reaches a certain level, the unfolded protein response (UPR) is activated [27,28], and if the UPR is not effective in relieving ER stress, prolonged stress may lead to pancreatic β-cell dysfunction and even cell death [27,29].

To investigate the compensatory enhanced secretion of insulin, we also observed a significant increase in the number of docked insulin granules in response to prolonged epinephrine stimulation (Figure 6B). The docking and fusion of insulin particles with the plasma membrane is an essential step in the insulin exocytosis process, in which soluble N-ethylmaleimide-sensitive factor attachment protein receptors (SNAREs) play a crucial role [30]. Our transcriptomic data also revealed that Syntaxin-2 (Stx2), a SNARE protein, was impaired following prolonged epinephrine exposure (Figure 4A). The flipping of Stx2 can modulate its inhibitory effect on insulin granule exocytosis, thereby affecting insulin secretion [31]. Thus, the impairment of Stx2 induced by epinephrine potentially disrupted insulin granule exocytosis.

Through the cell growth curves and cell cycle (Figure 2 and Figure 7D), we found that cell number and viability was consistently inhibited throughout the study period, even with the removal of epinephrine. This result supports previous findings that adrenergic signaling inhibits MIN6 cell proliferation [21]. However, how epinephrine affects β-cell survival through its specific receptors has not been thoroughly investigated. In this study, we found that under chronic epinephrine condition, MIN6 cells induced the expression of many genes known to be involved in endoplasmic reticulum processing, including key differential genes such as *CHOP*, *IRE1*, *ATF4*, *BiP*, and *XBP* (Figure 4A). The activation of *CHOP*, a classical proapoptotic protein in ER stress, is an important response to the *PERK*, *ATF6*, and *IRE1* activation of important proapoptotic signals [32,33,34]. Under ER stress, the activation of these three sensor proteins can lead to the initiation of UPR, in which the upregulation of *CHOP* is a vital step in the promotion of apoptosis, which promotes cells towards apoptosis by affecting the expression of several apoptosis-related genes, especially when ER stress remains unalleviated [29,35]. Therefore, we examined the gene expression levels of the PERK-ATF4-CHOP and IRE1α-XBP1s pathways, the activation of which reflects the cellular response to chronic epinephrine, leading to cellular dysfunction and apoptosis.

Reactive oxygen species (ROS) are highly reactive molecules that can impair β-cell function by decreasing insulin secretion, increasing insulin resistance, and altering gene expression. Oxidative stress and ER stress are highly interrelated biological processes that coexist and induce each other [36]; during ER stress, oxidative protein folding load and capacity are enhanced, leading to increased ROS production [37,38,39]. However, the endoplasmic reticulum antioxidant enzyme system has limited protective effects, which predisposes the intracellular oxidative and antioxidant systems to become imbalanced under ER stress in favor of an oxidized state, which in turn induces oxidative stress [38,40,41]. In the oxidative stress state, the levels of intracellular antioxidant enzymes are increased to adapt to the oxidative stress (Figure 8A–D). Consistent with previous studies, decreased expression of antioxidant genes due to *NRF2* downregulation (Figure 8E), as well as decreased *UCP2*, induces ROS accumulation (Figure 5B), which in turn leads to oxidative damage and cytotoxicity in β-cells. In addition, elevated expression levels of *OPA1*, *Pink1*, and *PRKN* were also detected, which may imply mitochondrial damage (Figure 5C). Thus, ROS production is associated with UPR under ER stress and induces the mitochondria to produce ROS as well, leading to oxidative stress, and oxidative stress predominantly arises from an imbalance between the generation of ROS and the protective mechanisms afforded by the corresponding antioxidants. In conclusion, prolonged exposure to epinephrine leads to increased ROS production. Despite the upregulation of antioxidant enzyme activities, the persistence of oxidative stress impairs the function and viability of pancreatic β-cells.

## 4. Materials and Methods

### 4.1. Cell Culture

MIN6 cells were originated from Prof. Jun-ichi Miyazaki (Osaka University, Japan) and were transferred from the West China Medical College of Sichuan University. Epinephrine for cell culture was obtained from the Animal Hospital of Southwest University, and the concentration used in this study was 100 nM. The experiments were conducted on MIN6 cells, with between 32 and 40 passages. The cells were grown in RPMI 1640 medium (Solarbio, Beijing, China), supplemented with 10% FBS (Gibco) and 50 µM β-mercaptoethanol (Gibco) in a humidified atmosphere of 5% CO_2_ at 37 °C.

### 4.2. Cell Number, Cell Viability, and Cycle Assays

Cell count: The procedure commenced with the detachment of cells utilizing Trypsin-EDTA, followed by their resuspension in a medium integrated with 0.4% Trypan Blue at a concentration of 10%. Subsequently, the cell suspension was transferred to a conventional hemocytometer for microscopic analysis at 10-fold magnification. To ensure accuracy, the enumeration process involved triplicate assessments of cell counts within a delineated grid. The arithmetic mean of these counts was then computed to ascertain the viable cell concentration per milliliter.

Cell viability assay: Cells (1 × 10^4^ cells/well) were inoculated in 96-well plates, and 100 μL of the compounds were added to fresh medium and incubated for 24 h. The cell culture medium was removed, 10% CCK8 (Beyotime, Shanghai, China) solution was applied to the medium, and it was reincubated for 2 h. The absorbance at 450 nm was measured using a microplate reader (Bio-Rad, Hercules, CA, USA).

Cell cycle assay: The cells were fixed using pre-cooled 70% ethanol at 4 °C for 24 h, followed by the addition of 0.5 mL of propidium iodide staining solution (Beyotime) in a warm bath at 37 °C, away from light, for 30 min. Red fluorescence was detected using a flow cytometer (ACEC) at an excitation wavelength of 488 nm, and data were processed using FlowJo software v10.

### 4.3. Insulin Secretion Assay

Glucose-stimulated insulin secretion was achieved in MIN6 cells, as described previously [5]. Briefly, MIN6 cells were incubated in Kreb’s buffer (118 mM NaCl, 4.8 mM KCl, 1.2 mM MgSO_4_, 1.2 mM KH_2_PO_4_, 2.5 mM NaHCO_3_, 2.5 mM CaCl_2_·2H_2_O (pH 7.4)) for half an hour, and then in Kreb’s buffer containing 0, 2.8, 10, and 20 mM glucose for 1 h, and the supernatant was removed for insulin assay. The cells were lysed with cold lysis buffer, and the total protein content was determined by BCA protein assay (Beyotime). The insulin content was measured using an insulin radioimmunoassay kit (BNIBT, F01TB) and normalized to the total protein content.

### 4.4. RNA Sequencing and Analysis

After different treatments, MIN6 cells (control, n = 3; chronic epinephrine exposure, n = 3) were collected and submitted to Gene Denovo Biotechnology (Guangzhou, China) for high-throughput RNA sequencing (RNA-seq). Briefly, total RNA was extracted using a Trizol kit (Invitrogen, Carlsbad, CA, USA), according to the manufacturer’s experimental protocols, and RNA integrity was assessed on a NanoPhotometer spectrophotometer and an Agilent 2100 Bioanalyzer system (Agilent Technologies, Santa Clara, CA, USA) to assess RNA integrity, and the resulting cDNA libraries were sequenced using an Illumina Novaseq6000. Statistical analysis was performed using DESeq2 and edgeR [42,43]. The GO and Kyoto Encyclopedia of Genes and Genomes (KEGG) terms, with corrected *p* < 0.05, were defined as significantly enriched by the commonly expressed genes (CEGs) and differently expressed genes (DEGs) [44].

### 4.5. Biochemical Parameters Assays

The samples were collected and centrifuged briefly to obtain a clear supernatant for analysis. Total antioxidant capacity (T-AOC assay kit, A015-3), superoxide dismutase box (SOD assay kit, A001-3-2), catalase (CAT assay kit, A007-1), glutathione peroxidase (GSH-Px assay kit, A005-1-2), and ATP content (ATP assay kit, A095-1-1) were assayed, in accordance with the production instructions of Nanjing Jiancheng Bioengineering Institute (Nanjing, China).

### 4.6. Transmission Electron Microscopy (TEM)

Well-treated cells were fixed using 2.5% glutaraldehyde at 4 °C for 4 h (Servicebio) and submitted to Servicebio Biotechnology (Wuhan, China) for ultrathin sectioning (70 nm) and transmission electron microscopy imaging (HITACHI HT7700). For quantification of the docked insulin granules, at least six random sections were used per group, and vesicles with dense bodies within 0.2 μm of the plasma membrane were considered to be docked insulin granules.

### 4.7. Quantitative Real Time PCR

Total RNA was extracted from MIN6 cells using Trizol reagent (Thermo Fisher Scientific, Waltham, MA, USA), and mRNA was reverse transcribed into cDNA using PrimeScript^TM^ RT reagent and gDNA Eraser (RR047A, Takara, Beijing, China). For each sample, TB Green Premix Ex Taq^TM^ II (RR820A, Takara, Beijing, China), combined with CFX96 Touch^TM^ Real-time qPCR Detection system (Bio-Rad, Hercules, CA, USA), was used to quantify the relative expression of mRNA. The primer sequences are shown in Table S1.

### 4.8. Statistical Analysis

Quantitative data were expressed as mean ± standard error of the mean (SEM). Statistical analysis was performed using GraphPad Prism 8.0.2 software. The statistical significance of the difference between the groups was determined by *t*-test, and the difference was statistically significant at *p* < 0.05. The data were from at least three biological replicates, and “n” represents the number of independent samples in each group. * *p* < 0.05; ** *p* < 0.01.

## 5. Conclusions

**Figure 9:**
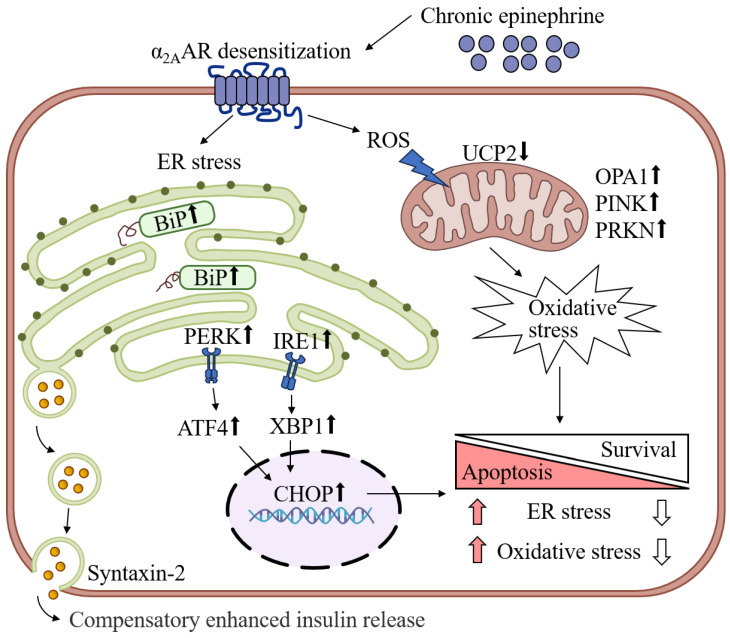
Summary of the effects of chronic epinephrine exposure on MIN6 cell function and fate.

Our research reveals profound effects of chronic epinephrine exposure on MIN6 cell function. Through high-throughput RNA sequencing, we identified multiple differentially expressed genes whose changes point to the regulation of key biological processes, including insulin secretion, cell fate, and oxidative stress. In particular, the inability of pancreatic β-cells to recover from prolonged adrenergic stimulation leads to the development of ER and oxidative stress, which impair islet β-cell function and survival (Figure 9). Furthermore, our data suggest a complex interaction between α2A-AR desensitization and the regulation of insulin secretion.

## Data Availability

The RNA-seq data is accessible at Gene Expression Omnibus (accession number: GSE269666). All remaining data are included in the article and Supplementary Materials files or are available from the authors upon reasonable request.

## Supplementary Materials

The following supporting information can be downloaded at: https://www.mdpi.com/article/10.3390/ijms25137029/s1.
