# Supplementary material for: Chronic Epinephrine-Induced Endoplasmic Reticulum and Oxidative Stress Impairs Pancreatic β-Cells Function and Fate
Source: Int J Mol Sci. 2024 Jun 27;25(13):7029. doi: 10.3390/ijms25137029 (PMC11241606; doi:10.3390/ijms25137029)
Supplement: Supplementary file 1 [file ijms-25-07029-s001.zip › ijms-3025480-supplementary.pdf]

Table S1. Primers for RT-qPCR

Gene	Forward	Reverse
α2A-AR	GGTGTGTTGGTTCCCGTTCT	CGGAAGTCGTGGTTGAAAATG
β-actin	GTGGGTATGGGTCAGAAGGAC	TGTGGTGCCAAATCTTCTCCA
ATF4	GTGGCCAAGCACTTGAAACCTCAT	TCCAACGTGGTCAAGAGCTCATCT
BiP	ACCAGGATGCGGACATTGAAGACT	AAAGCAGTAAACAGCCACTTGGGC
CCND1	GAACTACCTGGACCGCTTCC	CTCCTTCATCTTAGAGGCCACG
CTNNB1	ACTTGCCACACGTGCAATTC	CATGGTGCGTACAATGGCAGA
CHOP	GCGACAGAGCCAGAATAACA	GATGCACTTCCTTCTGGAACA
GADD34	TACCCGGAGAGAAGCCAGAA	GGCTTCGATCTCGTGCAAAC
IRE1α	CCTACAAGAGTATGTGGAGC	GGTCTCTGTGAACAATGTTGAGAG
NRF2	AGTCCCAGCAGGACATGGATTTGA	CTTGTTTGGGAATGTGGGCAACCT
OPA1	TCTTCACTGCAGGTCCCAAAT	TCTGACACCTTCCTGTAATGCTTG
PGC1α	CAACAATGAGCCTGCGAACA	GCATCAAATGAGGGCAATCC
PINK1	GTGGGACTCAGATGGCTGTC	CAGCCCAGGTATCGGCTTTG
PRKN	GGTCGATTCTGACACCAGCA	TGAACCGTCAGGTGATTCGG
PDX1	GAACGCTGGAACAGGGAAGT	CCAGTCTCGGTTCCATTCG
UCP2	CTGGGCACCATCCTAACC	GGAAGCGGACCTTTACCA
XBP1s	GAGTCCGCAGCAGGTG	GTGTCAGAGTCCATGGGA
XBP1u	AACACGCTTGGGAATGGACA	ACATAGTCTGAGTGCTGCGG
